# Safety and Efficacy of Tranexamic Acid in Aneurysmal Subarachnoid Hemorrhage: A Meta-Analysis of Randomized Controlled Trials

**DOI:** 10.3389/fneur.2021.710495

**Published:** 2022-01-24

**Authors:** Junwei Ren, Dongxi Qian, Jiaming Wu, Lingyan Ni, Wei Qian, Guozheng Zhao, Chuanjun Huang, Xing Liu, Yu Zou, Weikang Xing

**Affiliations:** ^1^Department of Neurosurgery, Suzhou Ninth People's Hospital, Suzhou, China; ^2^Department of Gastroenterology, Dushu Lake Hospital Affiliated to Soochow University, Suzhou, China; ^3^Department of Neurology, The First People's Hospital of Taicang, Suzhou, China

**Keywords:** tranexamic acid, aneurysmal subarachnoid hemorrhage, safety, efficacy, randomized controlled trials, meta-analysis

## Abstract

**Background:**

In recent decades, tranexamic acid (TXA) antifibrinolytic therapy before aneurysm clipping or embolization has been widely reported, but its safety and efficacy remain controversial. This meta-analysis evaluated the efficacy and safety of TXA therapy in aneurysmal subarachnoid hemorrhage (aSAH) patients, aiming to improve the evidence-based medical knowledge of treatment options for such patients.

**Methods:**

Pubmed, Web of Science, and Cochrane Library databases were searched up to 1 March 2021 for randomized controlled trials (RCTs). We extracted safety and efficacy outcomes and performed a meta-analysis using the Review Manager software. We performed two group analyses of TXA duration and daily dose.

**Results:**

Ten RCT studies, enrolling a total of 2,810 participants (1,410 with and 1,400 without TXA therapy), matched the selection criteria. In the TXA duration group: TXA did not reduce overall mortality during the follow-up period [RR 1.00 (95% CI 0.81–1.22)]. The overall rebleeding rate in the TXA group was 0.53 times that of the control group, which was statistically significant [RR 0.53 (95% CI 0.39–0.71)]. However, an RR of 0.43 was not statistically significant in the subgroup analysis of short-term therapy [RR 0.43 (95% CI 0.13–1.39)]. The overall incidence of hydrocephalus was significantly higher in the TXA group than in the control group [RR 1.13 (95% CI 1.02–1.24)]. However, the trend was not statistically significant in the subgroup analysis [short-term: RR 1.10 (95% CI 0.99–1.23); long-term: RR 1.22 (95% CI 0.99–1.50)]. Treatment with TXA did not cause significant delayed cerebral ischemia [RR 1.18 (95% CI 0.89–1.56)], and its subgroup analysis showed an opposite and insignificant effect [short-term: RR 0.99 (95% CI 0.79–1.25); long-term: RR 1.38 (95% CI 0.86–2.21)]. Results in the daily dose group were consistent with those in the TXA duration group.

**Conclusions:**

Tranexamic acid does not reduce overall mortality in patients with aSAH, nor does it increase the incidence of delayed cerebral ischemia. Tranexamic acid in treating aSAH can reduce the incidence of rebleeding. However, there is no statisticalsignificance in the ultra-early short-term and low daily dose TXA therapy, which may be due to the lack of relevant studies, and more RCT experiments are needed for further study.

**Systematic Review Registration:**

https://www.crd.york.ac.uk/PROSPERO/display_record.asp? PROSPERO, identifier: 244079.

## Introduction

Aneurysm subarachnoid hemorrhage (aSAH) accounts for 5% of all strokes and has an incidence of 7.9 per 100,000 person-years (1). The case fatality rate is ~35% due to initial hemorrhage or subsequent complications. Only 25% of the survivors have a good prognosis (2). In recent decades, tranexamic acid (TXA) antifibrinolytic therapy before aneurysm clipping or embolization has been widely reported, but its safety and efficacy remain controversial (3–6).

Rebleeding from the ruptured aneurysm increases the risk of poor outcomes and all-cause mortality (7). TXA can eliminate fibrinolysis in patients with SAH (8). At the end of the last century, many clinical studies on randomized controlled trials (RCTs) reported that TXA could significantly reduce the incidence of rebleeding in aSAH patients (5, 6, 9–11). Due to the conditions, they could not use TXA in the ultra-early stage for all patients. Moreover, simultaneously, those RCTs were mainly through the long-term (throughout the entire hospitalization) use of TXA. Although long-term antifibrinolytic therapy showed a reduction in rebleeding, the positive clinical outcome was negated by a concomitant rise in delayed cerebral ischemia (DCI) (4). However, most of all rebleeding in aSAH occur within the first 24 h (12). In 2002, Hillman J et al. found it by using TXA ultra-early (within 24 h) and for short-term (up to 72 h), a significant reduction in the rebleeding rate from 10.8 to 2.4% and an 80% reduction in the mortality rate from early rebleeding, without increasing the incidence of delayed ischemic neurological deficits (5). Unfortunately, it did not effectively improve overall mortality or clinical outcomes at 6 months (5).

With the development of diagnostic, therapeutic techniques, and materials, patients with aSAH are identified earlier and treated more successfully. Post et al.'s study, just published in 2021, had the most significant number of participants compared to any other study (13). From onset to first hospital contact for aSAH patients, the time was only 1.5 h, compared with more than 4 h in other trials (5, 6). They used the minimum daily dose of TXA (<4 g/d) for up to 24 h, greatly reducing the effects of delayed cerebral ischemia due to TXA. They noted that TXA might not significantly improve the incidence rate of rebleeding or better patient outcomes. These results were somewhat controversial with previous studies. Thus we produced this meta-analysis to evaluate the efficacy and safety of TXA therapy in aSAH patients.

## Methods

### Search Strategy

This meta-analysis was performed using the methodology recommended by the PRISMA (Preferred Reporting Items for Systematic Reviews and Meta-Analyses) guidelines. Two investigators independently conducted a systematic literature search through 1 March 2021 in the following databases: PubMed, Web of Science, and Cochrane Library. The terms used were “tranexamic acid” AND “subarachnoid hemorrhage OR intracranial aneurysm.” No language limitation was imposed in this study. The citations of identified articles were also filtered for additional studies.

### Selection Criteria

Studies meeting all the following inclusion criteria were considered eligible: (1) Patients participating in the trial were diagnosed with aSAH. (2) Patients in the treatment group received TXA treatment and conventional treatment. (3) The control group received conventional treatment without TXA. (4) Total mortality or good outcome probability and complications were reported. (5) It has to be an RCT study; the study quality needs to be high enough, meaning a Jadad score of 3–5 (ranging from 0 to 5) (14).

Studies need to be excluded: (1) Patients with traumatic SAH or spontaneous intracranial hemorrhage due to hypertension, arteriovenous malformation, and other causes. (2) TXA and placebo were not strictly prescribed in the treatment and control groups. (3) The study did not report primary or secondary outcomes or complications. (4) Not an RCT study or a Jadad score <3.

### Outcomes

The primary efficacy analysis was total mortality at the end of follow-up. The secondary efficacy endpoint was good outcome probability. The score of the modified Rankin Scale <3 was defined as a good outcome (15). Adverse events were used to assess the safety endpoints. Serious adverse events were defined as: rebleeding, hydrocephalus, and DCI during hospital admission.

### Data Collection

The essential information was extracted carefully and independently from each included study by two investigators: the first author's name, year of publication, country of research, the age range of participants, number of participants (with/without TXA), the latest time to use TXA, maximum duration usage of TXA, number of mortality, number of a good outcome and adverse events. We resolved any disagreement through discussion.

### Subgroup Analysis

We performed two group analyses of TXA duration and daily dose. The inclusion criteria for each group were as follows: (i) TXA duration group: (a) short-term TXA therapy: the treatment was administered immediately after diagnosing an aSAH and was pursued no longer than 72 h; (b) long-term TXA therapy: the treatment duration exceeding 72 h. (ii) TXA daily dose group: (a) low daily dose: TXA dose ≤ 4 g/d (grams per day); (b) high daily dose: TXA dose ≥6 g/d. According to this criterion, because of the TXA daily dose variation (6 g/d in the first week, 4 g/d in second to fifth weeks, 3 g/d in the sixth week), Fodstad H's study could not be included in any subgroup (10).

### Risk of Bias

The risk of bias plot was evaluated based on the Review Manager 5.4.1 software for each study. The unified standard of the Cochrane Collaboration was applied to assess the risk of bias of RCTs, which included: selection bias, performance bias, detection bias, attrition bias, reporting bias, and other biases.

### Statistical Analysis

Review Manager 5.4.1 software was used to conduct the analysis. Forest plot was performed by the pooled relative risks (RRs) with their 95% confidence intervals (CIs). Cochrane's *Q*-test and *I*^2^ were used to evaluate the statistical heterogeneity of the pooled results. The heterogeneity was considered to be significant when *p* < 0.05 (16). The *I*^2^ test with results ranging from 0 to 100% (*I*^2^ <25%, low heterogeneity; *I*^2^ = 25–50%, medium heterogeneity; *I*^2^ > 50%, high heterogeneity) was used to estimate the extent of heterogeneity better (17). The random-effects model was used to pool RRs in this meta-analysis.

## Results

### Literature Selection

**Figure 1:**
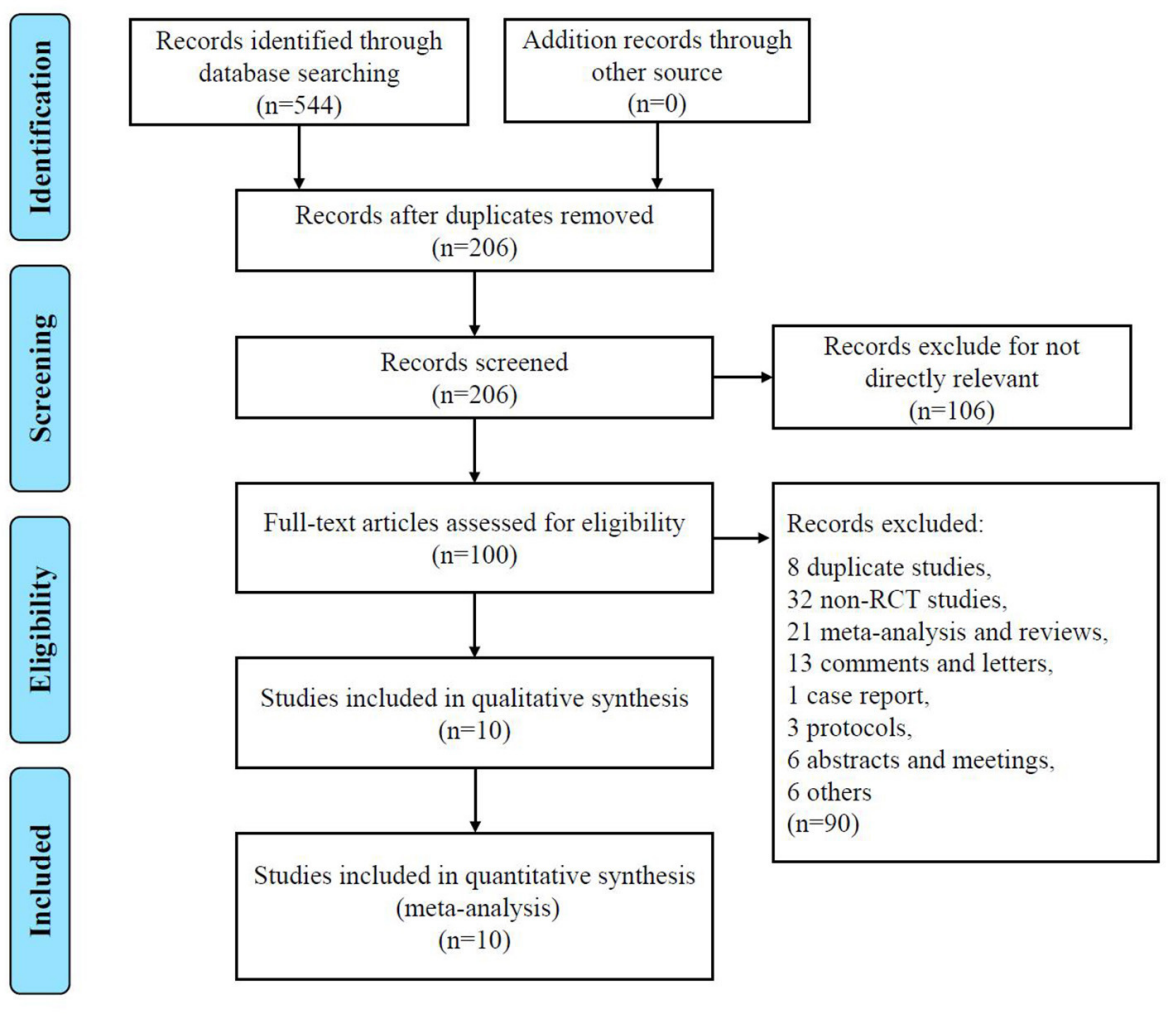
PRISMA flowchart detailing data screening and extraction PRISMA Preferred Reporting Items.

Figure 1 shows the PRISMA flow diagram of study selection. The search strategy identified 206 relevant articles screened in the PubMed, Web of Science, and Cochrane Library databases. We excluded 106 unrelated themes and had 100 articles for detailed evaluation. A total of 90 studies were excluded after further reading the full text. Twenty-one meta-analyses or reviews were excluded directly. Thirteen studies were comments or letters, one study was a case report, three were protocol articles, and six were abstract or meeting articles. Eight studies were excluded because of trial or data duplication (18–26). The remaining 6 studies were excluded due to indirectly controlled trials of TXA or lack of valuable data. Thus, a total of 10 studies were included in this meta-analysis (5, 6, 9–11, 13, 21, 27–29).

### Baseline Characteristics

The characteristics of the 10 included studies are shown in Table 1. There were 2,810 participants (1,410 with and 1,400 without TXA therapy) in the 10 studies. Patients in the study ranged in age from 15 to 73. Most of the researches were done in Europe, just one in Indonesia. Two studies were conducted on short-term treatment with TXA for subsequent subgroup analysis (5, 13). Earlier studies focused on a long-term therapy, up to 6 weeks. Table 2 shows detailed statistical information, including the number of deaths, good outcomes, rebleeding, hydrocephalus, and DCI.

**Table 1:** Characteristics of included studies.

**References**	**Country of research**	**Study period**	**Number of participants (male)**		**Age (years)**	**TXA dose**	**Treatment admission time**	**Maximum duration of TXA**	**Jadad score**
			**TXA**	**Control**					
Post et al. (13)	The Netherlands	July 24, 2013 to July 29, 2019	480 (148)	475 (163)	≥18	<4 g/d	Within 24 h	24 h	4
Hillman et al. (5)	Sweden	September 1997 to March 2000	254 (88)	251 (87)	≥15	4 g/d	Within 48 h	72 h	3
Roos (6)	The Netherlands	before December 31,1997	229 (89)	233 (72)	≥18	6 g/d	Within 96 h	3 weeks	5
Tsementzis et al. (21)	U.K.	February 1982 to September 1983	50 (20)	50 (26)	18–67	9 g/d	Within 72 h	4 weeks	5
Vermeulen et al. (9)	The Netherlands and U.K.	November 1977 to December 31 1982	241 (95)	238 (94)	NA	6 g/d	Within 72 h	4 weeks	5
Fodstad (10)	Sweden	1972 to 1978	53 (NA)	52 (NA)	19–73	6 g/d, (1 week) 4 g/d, (2–5 weeks) 3 g/d, (6 week)	Within 72 h	6 weeks	3
Kaste and Ramsay (27)	Finland	Before 1979 (NA)	32 (16)	32 (14)	11–60	6 g/d	Within 72 h	3 weeks	4
Maurice-Williams (28)	U.K.	September 1974 to May 1977	25 (NA)	25 (NA)	<65	6 g/d	Within 96 h	6 weeks	3
Chandra (11)	Indonesia	January 1, 1974 to January 1, 1976	20 (11)	19 (10)	20–65	6 g/d	Within 72 h	3 weeks	4
van Rossum et al. (29)	The Netherlands	November 1973 to November 1975	26 (NA)	25 (NA)	NA	4 g/d	Within 2 weeks	2 weeks	5

*U.K., the United Kingdom; NA, not applicable; H, hours*.

**Table 2:** Statistical information of included studies.

	**No. of mortality (total)**		**No. of good outcome (total)**		**No. of rebleeding (total)**		**No. of hydrocephalus (total)**		**No. of delayed cerebral ischemia (total)**	
**References**	**TXA**	**Control**	**TXA**	**Control**	**TXA**	**Control**	**TXA**	**Control**	**TXA**	**Control**
Post et al. (13)	128 (480)	114 (475)	229 (480)	262 (475)	42 (480)	57 (475)	292 (480)	262 (475)	108 (480)	106 (475)
Hillman et al. (5)	33 (254)	41 (251)	190 (254)	177 (251)	6 (254)	27 (251)	30 (254)	27 (251)	10 (254)	12 (251)
Roos (6)	NA	NA	115 (229)	128 (233)	44 (229)	77 (233)	71 (229)	62 (233)	68 (229)	74 (233)
Tsementzis et al. (21)	19 (50)	14 (50)	27 (50)	30 (50)	NA	NA	19 (50)	15 (50)	22 (50)	11 (50)
Vermeulen et al. (9)	84 (241)	89 (238)	127 (241)	126 (238)	21 (241)	56 (238)	35 (241)	29 (238)	59 (241)	36 (238)
Fodstad (10)	13 (53)	10 (52)	NA	NA	7 (53)	16 (52)	NA	NA	NA	NA
Kaste and Ramsay (27)	4 (32)	4 (32)	NA	NA	7 (32)	6 (32)	15 (32)	8 (32)	NA	NA
Maurice-Williams (28)	3 (25)	11 (25)	22 (25)	14 (25)	6 (25)	14 (25)	4 (25)	7 (25)	NA	NA
Chandra (11)	1 (20)	5 (19)	NA	NA	1 (20)	4 (19)	NA	NA	NA	NA
van Rossum et al. (29)	15 (26)	11 (25)	NA	NA	5 (26)	4 (25)	NA	NA	NA	NA

*No., number; NA, not applicable*.

### Efficacy Endpoints

#### Mortality

**Figure 2:**
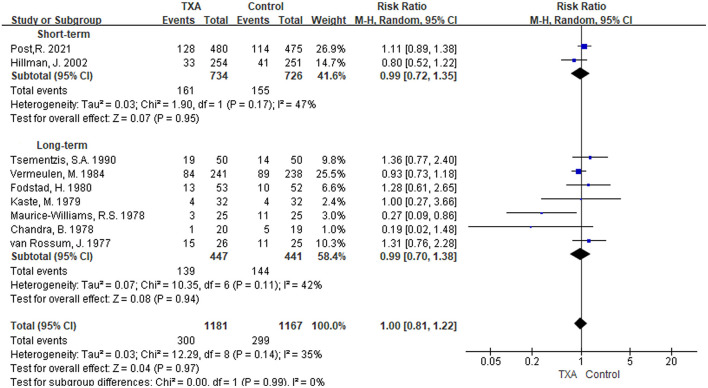
Forest plot of comparison in mortality. CI, confidence interval; df, degrees of freedom; TXA, tranexamic acid; M-H, Mantel-Haenszel.

i) TXA duration group: Nine studies reported mortality during the follow-up period (5, 9–11, 13, 21, 27–29). There was no significant difference in overall mortality, with a RR of 1.00 between the TXA and control groups [RR 1.00 (95% CI 0.81–1.22), *I*^2^ = 35%; Figure 2]. Again, no significant difference was found in the subgroup analysis of the duration to treatment [short-term: RR 0.99 (95% CI 0.72–1.35), *I*^2^ = 47%; long-term: RR 0.99 (95% CI 0.70–1.38), *I*^2^ = 42%; Figure 2].
ii) TXA daily dose group: Overall, eight studies that presented mortality during follow-up were included (5, 9, 11, 13, 21, 27–29). Similarly, there was no significant difference in overall mortality, with an RR of 0.97 [RR 0.97 (95% CI 0.78–1.22), *I*^2^ = 41%; Supplementary Figure 1]. No significant results were found in subgroup analysis of daily dose [low daily dose: RR 1.05 (95% CI 0.84–1.32), *I*^2^ = 21%; high daily dose: RR 0.81 (95% CI 0.49–1.35), *I*^2^ = 42%; Supplementary Figure 1].

### Good Outcome

**Figure 3:**
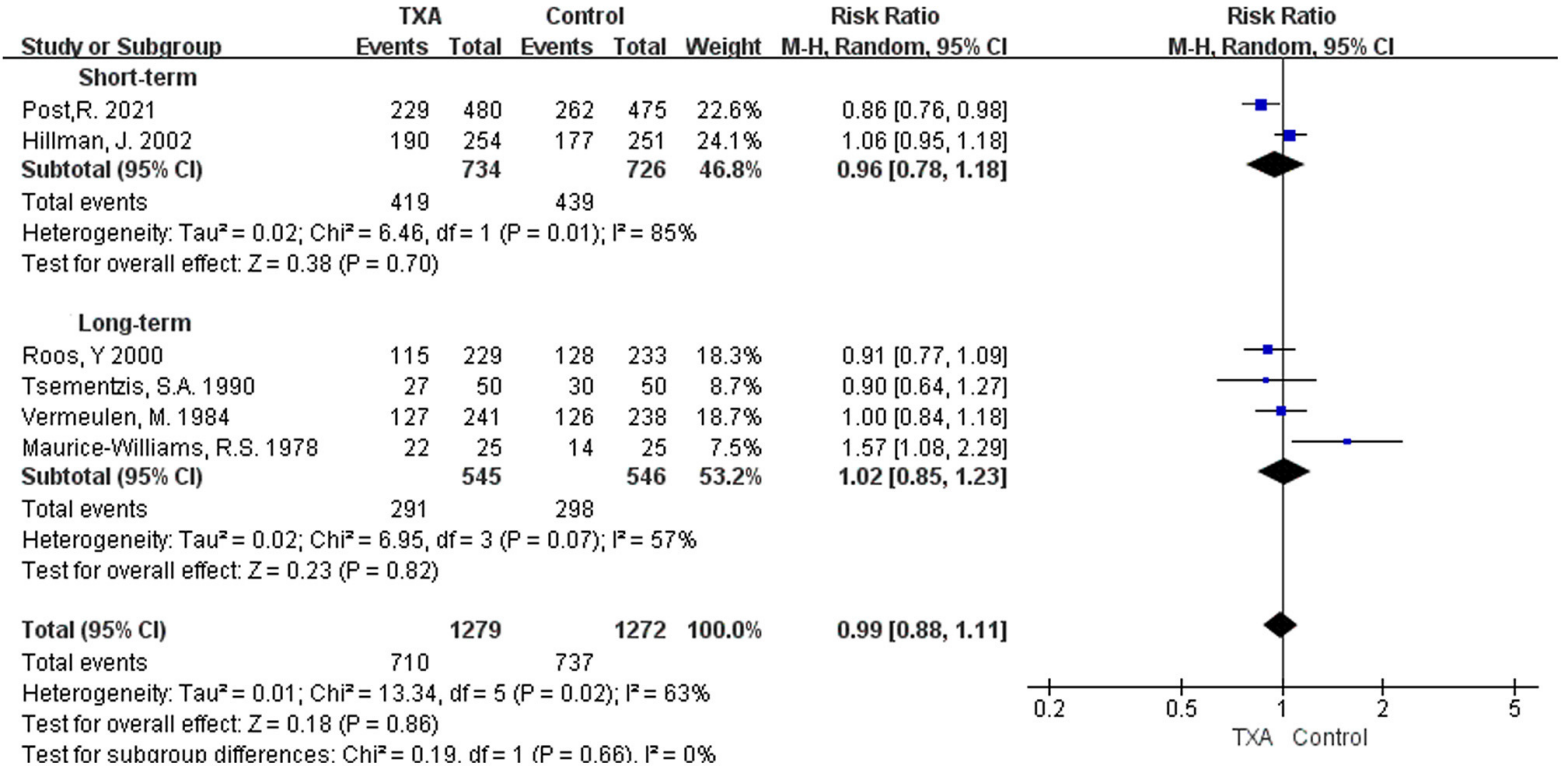
Forest plot of comparison in a good outcome. CI, confidence interval; df, degrees of freedom; TXA, tranexamic acid; M-H, Mantel-Haenszel.

i) TXA duration group: Six studies provided data on good outcome (5, 6, 9, 13, 21, 28). The pooled overall good outcome of these six studies was not significantly improved [RR 0.99 (95% CI 0.88–1.11), *I*^2^ = 63%; Figure 3]. In the subgroup analysis, TXA was not found to significantly promote good outcome in either the short or long-term treatment regimens [short-term: RR 0.96 (95% CI 0.78–1.18), *I*^2^ = 85%; long-term: RR 1.02 (95% CI 0.85–1.23), *I*^2^ = 57%; Figure 3].
ii) TXA daily dose group: In this subgroup, the included studies were the same as the TXA duration group, and the results were exact (Supplementary Figure 2).

### Safety Endpoints

#### Rebleeding

**Figure 4:**
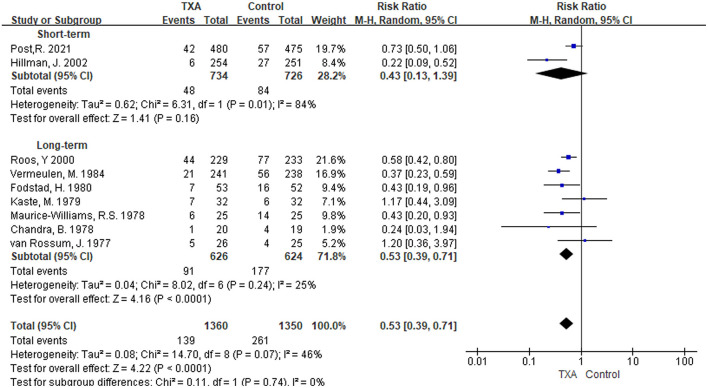
Forest plot of comparison in rebleeding. CI, confidence interval; df, degrees of freedom; TXA, tranexamic acid; M-H, Mantel-Haenszel.

i) TXA duration group: Rebleeding was reported in nine studies (5, 6, 9–11, 13, 27–29). We found that in the overall pooled outcome, the incidence of rebleeding was significantly reduced in the TXA treatment group compared with the control group [RR 0.53 (95% CI 0.39–0.71), *I*^2^ = 46%; Figure 4]. In the subgroup analysis, the pooled result of long-term TXA treatment also yielded a significant reduction in rebleeding [RR 0.53 (95% CI 0.39–0.71), *I*^2^ = 25%; Figure 4]. Unfortunately, although the rate of rebleeding was only 0.43 times that of the control group with short-term TXA therapy, it was not statistically significant [RR 0.43 (95% CI 0.13–1.39), *I*^2^ = 84%; Figure 4].
ii) TXA daily dose group: Eight studies with rebleeding results met the inclusion criteria (5, 6, 9, 11, 13, 27–29). We found a significant reduction in rebleeding in both the overall pooled outcome and the high-daily dose subgroup [overall: RR 0.54 (95% CI 0.38–0.74), *I*^2^ = 51%; high daily dose: RR 0.51 (95% CI 0.36–0.72), *I*^2^ = 32%; Supplementary Figure 3]. However, the low daily dose subgroup only reduced the tendency for rebleeding but was not significant [RR 0.56 (95% CI 0.23–1.35), *I*^2^ = 73%; Supplementary Figure 3].

#### Hydrocephalus

**Figure 5:**
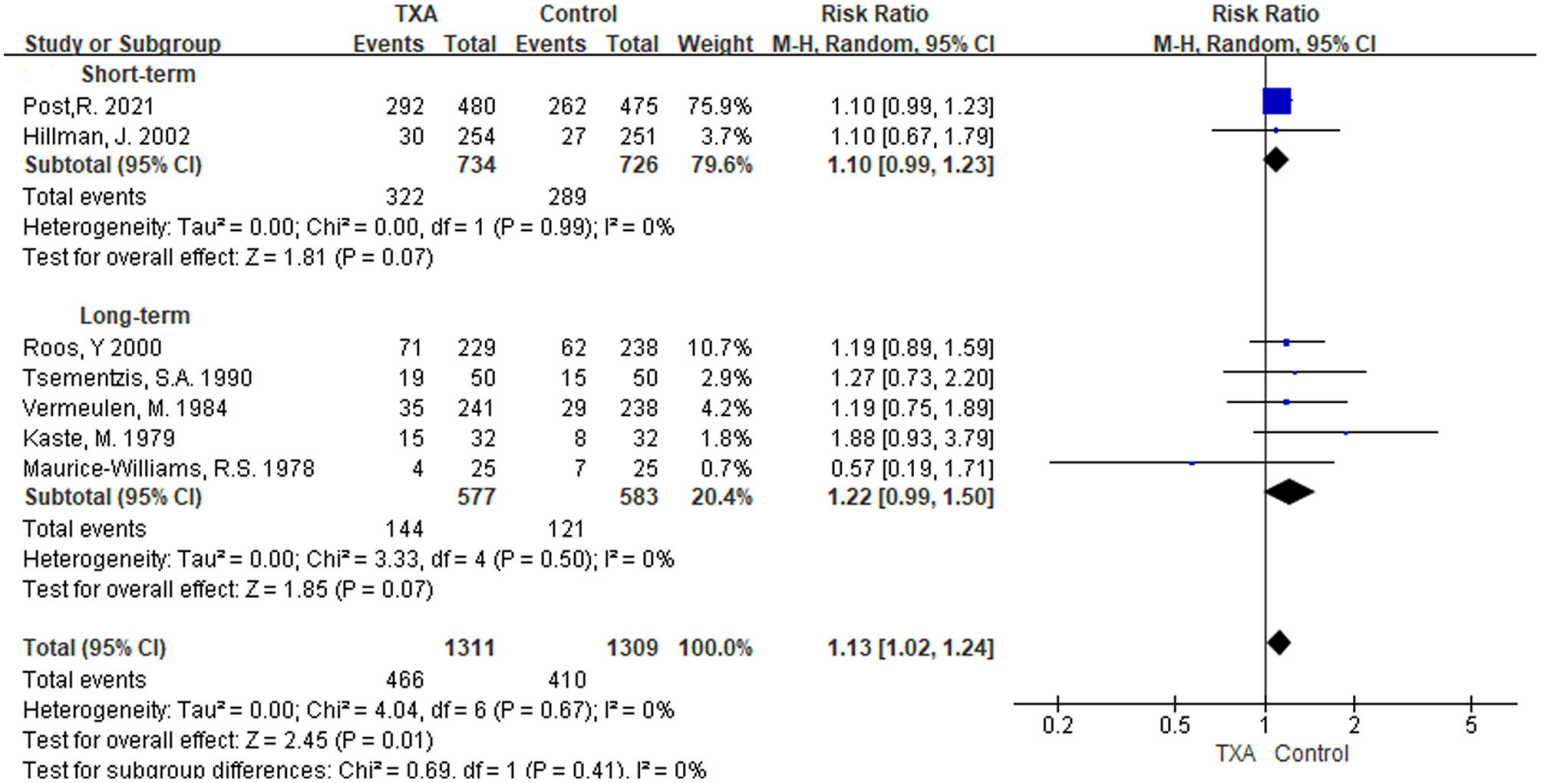
Forest plot of comparison in hydrocephalus. CI, confidence interval; df, degrees of freedom; TXA, tranexamic acid; M-H, Mantel-Haenszel.

i) TXA duration group: Seven studies referred to results on the incidence of hydrocephalus (5, 6, 9, 13, 21, 27, 28). As shown in Figure 5, although the pooled results in the two subgroup analyses were not statistically significant [short-term: RR 1.10 (95% CI 0.99–1.23), *I*^2^ = 0%; long-term: RR 1.22 (95% CI 0.99–1.50), *I*^2^ = 0%], the overall pooled results in the incidence of hydrocephalus were significantly higher [RR 1.13 (95% CI 1.02–1.24), *I*^2^ = 0%].
ii) TXA daily dose group: The included studies and outcomes were the same as the TXA duration group (Supplementary Figure 4).

#### Delayed Cerebral Ischemia

**Figure 6:**
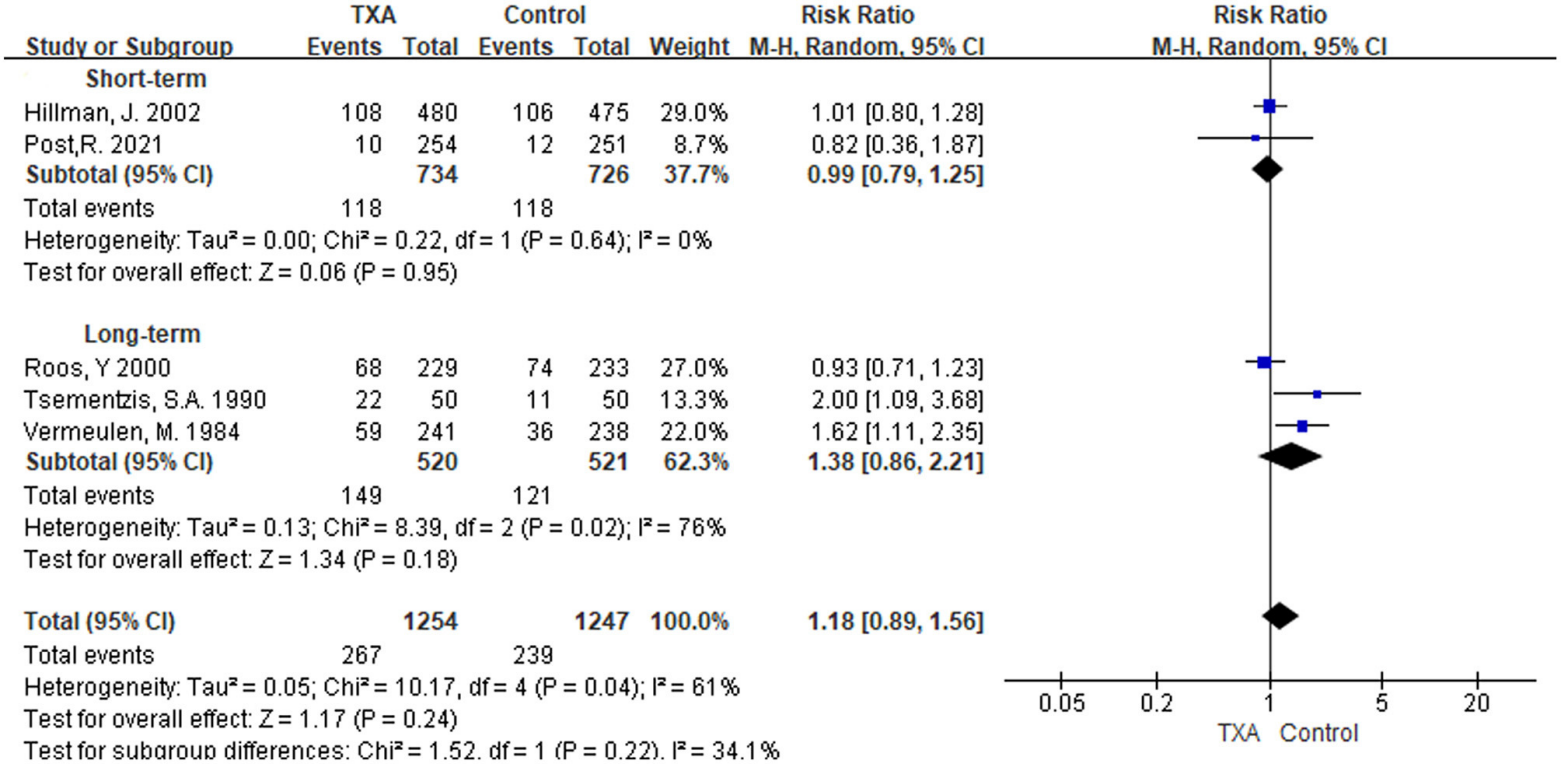
Forest plot of comparison in delayed cerebral ischemia. CI, confidence interval; df, degrees of freedom; TXA, tranexamic acid; M-H, Mantel-Haenszel.

i) TXA duration group: We found five studies that reported the condition of DCI (5, 6, 9, 13, 21). In the overall pooled result, the incidence of DCI in the TXA treatment group was 1.18 times higher than that in the control group, but it was not statistically significant [RR 1.18 (95% CI 0.89–1.56), *I*^2^ = 61%; Figure 6]. Short-term and long-term TXA therapy appeared to have opposite but not significant effects on the development of DCI [short-term: RR 0.99 (95% CI 0.79–1.25), *I*^2^ = 0%; long-term: RR 1.38 (95% CI 0.86–2.21), *I*^2^ = 76%; Figure 6].
ii) TXA daily dose group: The included studies and outcomes were the same as the TXA duration group (Supplementary Figure 5).

### Risk of Bias

**Figure 7:**
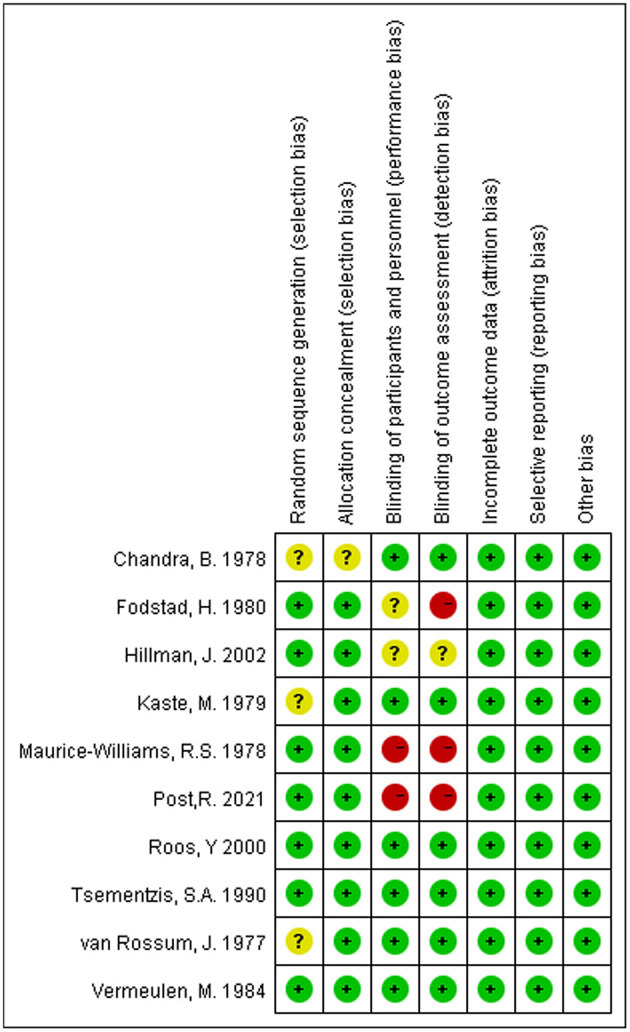
Risk of bias summary: Review authors' judgments about each risk of bias item for each included study.

Figure 7 shows the details of the risk bias for each included study. Three clinical trials had an unclear risk of bias in random sequence generation. For allocation concealment, the risk of bias was unclear in one study. Two trials were high risk for blinding participants and personnel, and two were unclear. For the blinding of outcome assessment, the risk of bias was high in three trials and unclear in one trial. No trial had an unclear or high risk of bias in incomplete outcome data, selective reporting, and other biases. Overall, the quality of the studies included in this meta-analysis was acceptable.

## Discussion

We performed a meta-analysis of 10 RCT studies, including the latest RCT published earlier this year by René Post et al. (13). The duration and the daily dose of TXA were analyzed as the subgroups. We found that TXA therapy tended to be long-term, high daily dose in some earlier studies, while it tended to be short-term, low daily dose in studies after 2000. Therefore, the groups according to duration or daily dose are generally consistent. Only van Rossum et al.'s study used a long-term medication with a low daily dose but had no effect on the outcomes of the two groups due to its small sample size and reported only rebleeding rates and mortality (29). Our meta-analysis found that long-term TXA therapy significantly reduced the incidence of rebleeding in aSAH patients. Both short- and long-term treatments failed to show a consistent, significant improvement in mortality and good outcome, despite a consistent reduction in rebleeding. Besides, TXA did not increase DCI occurrence but significantly generated hydrocephalus. The subgroup analysis of daily doses reached the same conclusion. A relatively new meta-analysis reported that TXA reduced the incidence of rebleeding without increasing the incidence of hydrocephalus (30). However, their study only included five research and did not conduct further subgroup analysis, leading to differences from our results. It should be reminded that one research included by them, Post et al. (18), was excluded from our study because of the research data duplication from the latest study published in 2021 (13, 18).

In our analysis, only long-term TXA therapy reduced the risk of rebleeding, with no significant reduction in the short term. Statistically, the risk of rebleeding within 12 h in aSAH patients is ~8% (31, 32). René Post et al. found in the latest study that TXA therapy may benefit patients the most in the time window since most rebleeding occurs within 24 h of the first hemorrhage, and antifibrinolytic therapy may aggravate cerebral ischemia. However, short-term (<24 h) TXA therapy did not significantly reduce rebleeding or improve outcomes at 6 months (13). With a median time from diagnosis to aneurysm treatment of 14 h, the benefit of this early aneurysm treatment may now outweigh the reduction in rebleeding of tranexamic acid. Jan Hillman et al. showed a different conclusion that the incidence of rebleeding decreased significantly, and only one patient in the TXA group had a rebleeding within 24 h. Nevertheless, unlike René Post's study, where TXA therapy was <24 h, nearly half of the patients in their study received TXA therapy for longer than 24 h (5). Among the five studies in Feng and Chen's meta-analysis (30), two of the long-term TXA therapy research suggested that TXA could reduce rebleeding (9, 29), while the other three short-term research did not (13, 18, 33). Notably, one of the short-term studies used a high daily dose of TXA (33). Therefore, the incidence of rebleedingmay be more correlated with the duration of antifibrinolytic therapy. Some earlier studies concluded no significant difference in the rebleeding rate between the experimental and control groups. The sample size of these earlier studies was small, and in one of them, the time window of TXA initial treatment for patients was extended to 2 weeks (11, 27, 29). Admittedly, these deficiencies may cause some bias in our results. Besides, cause there were few studies on ultra-early short-term treatment, it was unsure whether this treatment regimen has no preventive effect on rebleeding. More experiments on ultra-early short-term medication may also be needed to help the study.

Whether the TXA therapy will lead to DCI is also the focus of clinical attention. Our study showed that neither long-term nor short-term TXA therapy increased the incidence of DCI in aSAH patients. There was also no significant difference in the daily dose. Among the included studies, most of those using long-term and high daily dose TXA therapy believed that TXA would increase DCI incidence, while those using short-term and low daily dose TXA therapy believed that TXA would not. A previous study using short-term with high Daily Dose TXA therapy also found an increased incidence of DCI (33). In conclusion, the daily dose of TXA may be more closely related to the occurrence of DCI, but more studies needing for further evaluation. Although TXA can effectively reduce rebleeding in aSAH patients, it does not improve their prognosis (6, 9). It may be that cerebral ischemia induced by TXA offsets the benefits of reduced rebleeding (34). However, after the combination of nimodipine to alleviate cerebral ischemia caused by TXA, the prognosis of patients still did not improve significantly (35). The anti-vasospasm effect of calcium antagonists is probably insufficient to counteract the TXA-induced cerebral ischemia (6).

As a common complication of aSAH, hydrocephalus is usually caused by the accumulation of blood in the ventricle, affecting the reflux of cerebrospinal fluid (36). TXA reduces plasminogen activity in cerebrospinal fluid, leading to poor absorption of intraventricular hemorrhage, which is more likely to cause hydrocephalus (21, 36). Our results support this theory, finding that TXA significantly increased 13% hydrocephalus in aSAH patients. Of course, we expect more and more studies to be further analyzed.

Some clinical complications common in aSAH patients, such as epilepsy, transient ischemic attack, and delirium, may also be associated with TXA (37, 38). However, due to the lack of relevant reports, no further study was conducted in this meta-analysis.

## Limitation

Our meta-analysis also has some limitations. First, the majority of the included articles were European studies with a low selectivity of the population, which may have generated some bias. Second, most of the studies included were published decades ago, with a sizeable period. Although subgroup analysis is helpful, it does not offset the bias due to continuous advances in diagnosis, treatment, and materials. Third, we did not assess the impact of gender differences on outcomes. The majority of included studies showed a higher proportion of women, possibly because aneurysms are more common in the female population. However, this is the inevitable defect of this meta-analysis. Finally, the publication bias inherent in meta-analysis itself cannot be ignored. Considering the above limitations, we should cautiously interpret our results.

## Conclusion

In conclusion, TXA did not reduce overall mortality in aSAH patients, nor did it improve the incidence of a good outcome. It should be noted that TXA may cause an increase in hydrocephalus but not DCI. TXA reduced the incidence of rebleeding but was not statistical significance in the ultra-early short-term and low daily dose therapy. More RCT experiments are needed for further study.

## Data Availability Statement

The original contributions presented in the study are included in the article/Supplementary Material, further inquiries can be directed to the corresponding author.

## Author Contributions

WX was the major contributor regarding the design of the study. WX, JR, and DQ were responsible for the statistical analysis and writing the first manuscript. JR, DQ, JW, and LN were responsible for revising the manuscript, proofreading the collected data, and validating the included studies. WQ, GZ, CH, XL, and YZ contributed to data collection, plotting, and editing analysis tables and graphs. WX conceived the study and was in charge of overall direction and planning. All authors contributed to the article and approved the submitted version.

## Conflict of Interest

The authors declare that the research was conducted in the absence of any commercial or financial relationships that could be construed as a potential conflict of interest.

## Publisher's Note

All claims expressed in this article are solely those of the authors and do not necessarily represent those of their affiliated organizations, or those of the publisher, the editors and the reviewers. Any product that may be evaluated in this article, or claim that may be made by its manufacturer, is not guaranteed or endorsed by the publisher.
