# Supplementary material for: Safety and Efficacy of Tranexamic Acid in Aneurysmal Subarachnoid Hemorrhage: A Meta-Analysis of Randomized Controlled Trials
Source: Front Neurol. 2022 Jan 24;12:710495. doi: 10.3389/fneur.2021.710495 (PMC8818684; doi:10.3389/fneur.2021.710495)
Supplement: Supplementary file 1 [file Table_1.docx]

Supplementary table 1 Meta-analysis of various outcomes.

| **Endpoints** | **TXA duration group** | | | | | | **TXA daily dose group** | | | | | |
| --- | --- | --- | --- | --- | --- | --- | --- | --- | --- | --- | --- | --- |
|  | **Overall** | | **Short-term** | | **Long-term** | | **Overall** | | **Low daily dose** | | **High daily dose** | |
|  | **RR(95%CI)** | ***p* value** | **RR(95%CI)** | ***p* value** | **RR(95%CI)** | ***p* value** | **RR(95%CI)** | ***p* value** | **RR(95%CI)** | ***p* value** | **RR(95%CI)** | ***p* value** |
| Mortality | 1.00(0.81–1.22) | 0.97 | 0.99(0.72-1.35) | 0.95 | 0.99(0.70-1.38) | 0.94 | 0.97(0.78-1.22) | 0.82 | 1.05(0.84-1.32) | 0.67 | 0.81(0.49-1.35) | 0.32 |
| Good outcome | 0.99(0.88-1.11) | 0.86 | 0.96(0.78-1.18) | 0.70 | 1.02(0.85-1.23) | 0.82 | 0.99(0.88-1.11) | 0.86 | 0.96(0.78-1.18) | 0.70 | 1.02(0.85-1.23) | 0.82 |
| Rebleeding | 0.53(0.39-0.71) | <0.0001 | 0.43(0.13-1.39) | 0.16 | 0.53(0.39-0.71) | <0.0001 | 0.54(0.38-0.74) | 0.0002 | 0.56(0.23-1.35) | 0.20 | 0.51(0.36-0.72) | 0.0002 |
| Hydrocephalus | 1.13(1.02-1.24) | 0.01 | 1.10(0.99-1.23) | 0.07 | 1.22(0.99-1.50) | 0.07 | 1.13(1.02-1.24) | 0.01 | 1.10(0.99-1.23) | 0.07 | 1.22(0.99-1.50) | 0.07 |
| Delayed cerebral ischemia | 1.18(0.89-1.56) | 0.24 | 0.99(0.79-1.25) | 0.95 | 1.38(0.86-2.21) | 0.18 | 1.18(0.89-1.56) | 0.24 | 0.99(0.79-1.25) | 0.95 | 1.38(0.86-2.21) | 0.18 |

**TXA**: tranexamic acid. **RR**: risk ratio. **CI**: confidence interval.
