# Supplementary material for: Safety and Efficacy of Tranexamic Acid in Aneurysmal Subarachnoid Hemorrhage: A Meta-Analysis of Randomized Controlled Trials
Source: Front Neurol. 2022 Jan 24;12:710495. doi: 10.3389/fneur.2021.710495 (PMC8818684; doi:10.3389/fneur.2021.710495)
Supplement: Supplementary file 2 [file Image_1.pdf]

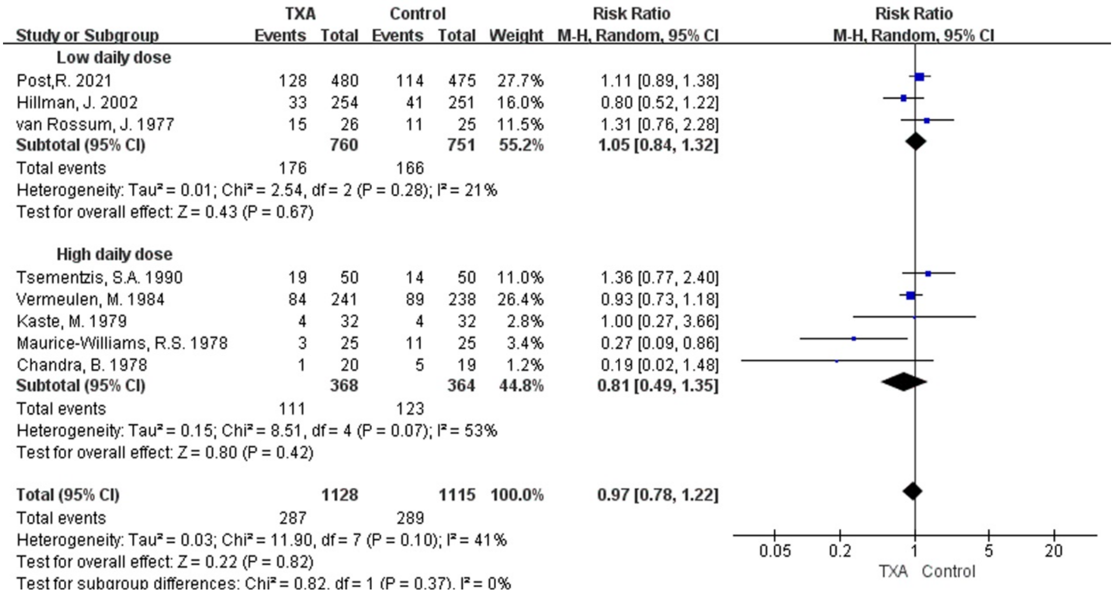

Supplementary figure 1. Forest plot of comparison in ***mortality****. CI = confidence interval, df = degrees of freedom, TXA = tranexamic acid, M-H = Mantel-Haenszel.*

Study or Subgroup	TXA Events	TXA Total	Control Events	Control Total	Weight	Risk Ratio M-H, Random, 95% CI
**Low daily dose**						
Post,R. 2021	229	480	262	475	22.6%	0.86 [0.76, 0.98]
Hillman, J. 2002	190	254	177	251	24.1%	1.06 [0.95, 1.18]
**Subtotal (95% CI)**		**734**		**726**	**46.8%**	**0.96 [0.78, 1.18]**
Total events	419		439			
Heterogeneity: Tau² = 0.02; Chi² = 6.46, df = 1 (P = 0.01); I² = 85%						
Test for overall effect: Z = 0.38 (P = 0.70)						
**High daily dose**						
Roos, Y 2000	115	229	128	233	18.3%	0.91 [0.77, 1.09]
Tsementzis, S.A. 1990	27	50	30	50	8.7%	0.90 [0.64, 1.27]
Vermeulen, M. 1984	127	241	126	238	18.7%	1.00 [0.84, 1.18]
Maurice-Williams, R.S. 1978	22	25	14	25	7.5%	1.57 [1.08, 2.29]
**Subtotal (95% CI)**		**545**		**546**	**53.2%**	**1.02 [0.85, 1.23]**
Total events	291		298			
Heterogeneity: Tau² = 0.02; Chi² = 6.95, df = 3 (P = 0.07); I² = 57%						
Test for overall effect: Z = 0.23 (P = 0.82)						
**Total (95% CI)**		**1279**		**1272**	**100.0%**	**0.99 [0.88, 1.11]**
Total events	710		737			
Heterogeneity: Tau² = 0.01; Chi² = 13.34, df = 5 (P = 0.02); I² = 63%						
Test for overall effect: Z = 0.18 (P = 0.86)						
Test for subgroup differences: Chi² = 0.19, df = 1 (P = 0.66), I² = 0%						

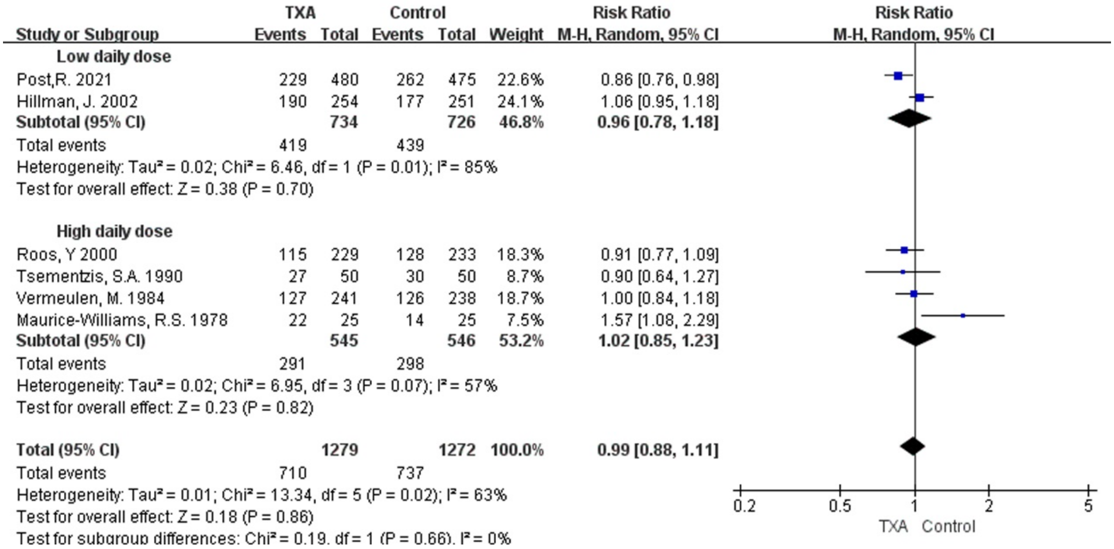

Supplementary figure 2. Forest plot of comparison in a ***good outcome****. CI = confidence interval, df = degrees of freedom, TXA = tranexamic acid, M-H = Mantel-Haenszel.*

Study or Subgroup	TXA Events	TXA Total	Control Events	Control Total	Weight	Risk Ratio M-H, Random, 95% CI
**Low daily dose**						
Post,R. 2021	42	480	57	475	21.2%	0.73 [0.50, 1.06]
Hillman, J. 2002	6	254	27	251	9.7%	0.22 [0.09, 0.52]
van Rossum, J. 1977	5	26	4	25	6.1%	1.20 [0.36, 3.97]
**Subtotal (95% CI)**		**760**		**751**	**37.0%**	**0.56 [0.23, 1.35]**
Total events	53		88			
Heterogeneity: Tau² = 0.43; Chi² = 7.52, df = 2 (P = 0.02); I² = 73%						
Test for overall effect: Z = 1.29 (P = 0.20)						
**High daily dose**						
Roos, Y 2000	44	229	77	233	22.9%	0.58 [0.42, 0.80]
Vermeulen, M. 1984	21	241	56	238	18.4%	0.37 [0.23, 0.59]
Kaste, M. 1979	7	32	6	32	8.3%	1.17 [0.44, 3.09]
Maurice-Williams, R.S. 1978	6	25	14	25	11.1%	0.43 [0.20, 0.93]
Chandra, B. 1978	1	20	4	19	2.3%	0.24 [0.03, 1.94]
**Subtotal (95% CI)**		**547**		**547**	**63.0%**	**0.51 [0.36, 0.72]**
Total events	79		157			
Heterogeneity: Tau² = 0.05; Chi² = 5.92, df = 4 (P = 0.21); I² = 32%						
Test for overall effect: Z = 3.79 (P = 0.0002)						
**Total (95% CI)**		**1307**		**1298**	**100.0%**	**0.54 [0.38, 0.74]**
Total events	132		245			
Heterogeneity: Tau² = 0.10; Chi² = 14.33, df = 7 (P = 0.05); I² = 51%						
Test for overall effect: Z = 3.71 (P = 0.0002)						
Test for subgroup differences: Chi² = 0.04, df = 1 (P = 0.84), I² = 0%						

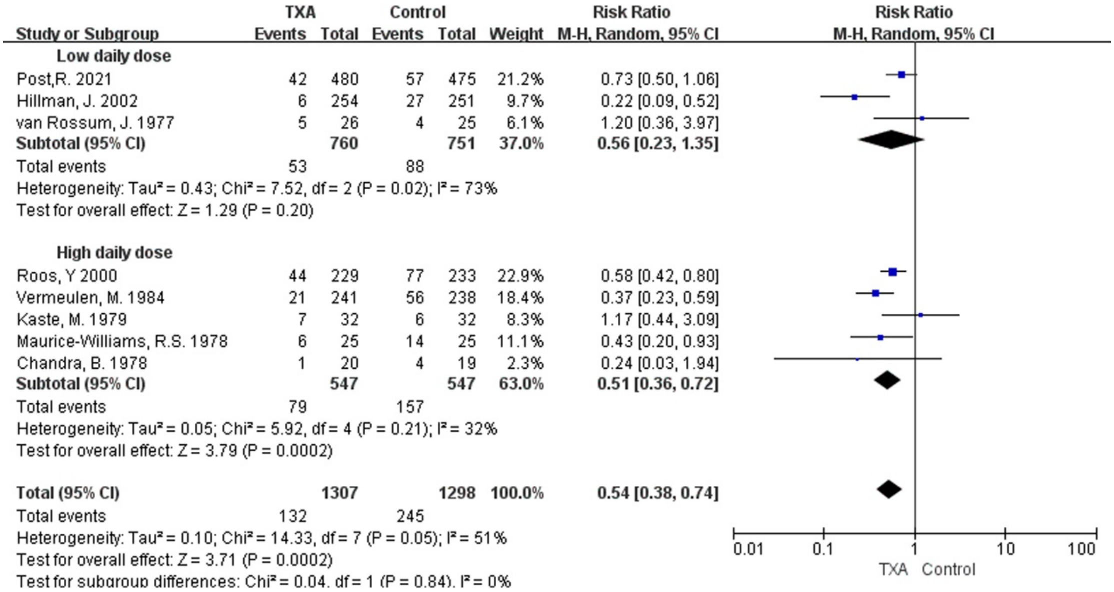

Supplementary figure 3. Forest plot of comparison in ***rebleeding****. CI = confidence interval, df = degrees of freedom, TXA = tranexamic acid, M-H = Mantel-Haenszel.*

Study or Subgroup	TXA Events	TXA Total	Control Events	Control Total	Weight	Risk Ratio M-H, Random, 95% CI
**Low daily dose**						
Post,R. 2021	292	480	262	475	75.9%	1.10 [0.99, 1.23]
Hillman, J. 2002	30	254	27	251	3.7%	1.10 [0.67, 1.79]
**Subtotal (95% CI)**		**734**		**726**	**79.6%**	**1.10 [0.99, 1.23]**
Total events	322		289			
Heterogeneity: Tau² = 0.00; Chi² = 0.00, df = 1 (P = 0.99); I² = 0%						
Test for overall effect: Z = 1.81 (P = 0.07)						
**High daily dose**						
Roos, Y 2000	71	229	62	238	10.7%	1.19 [0.89, 1.59]
Tsementzis, S.A. 1990	19	50	15	50	2.9%	1.27 [0.73, 2.20]
Vermeulen, M. 1984	35	241	29	238	4.2%	1.19 [0.75, 1.89]
Kaste, M. 1979	15	32	8	32	1.8%	1.88 [0.93, 3.79]
Maurice-Williams, R.S. 1978	4	25	7	25	0.7%	0.57 [0.19, 1.71]
**Subtotal (95% CI)**		**577**		**583**	**20.4%**	**1.22 [0.99, 1.50]**
Total events	144		121			
Heterogeneity: Tau² = 0.00; Chi² = 3.33, df = 4 (P = 0.50); I² = 0%						
Test for overall effect: Z = 1.85 (P = 0.07)						
**Total (95% CI)**		**1311**		**1309**	**100.0%**	**1.13 [1.02, 1.24]**
Total events	466		410			
Heterogeneity: Tau² = 0.00; Chi² = 4.04, df = 6 (P = 0.67); I² = 0%						
Test for overall effect: Z = 2.45 (P = 0.01)						
Test for subgroup differences: Chi² = 0.69, df = 1 (P = 0.41), I² = 0%						

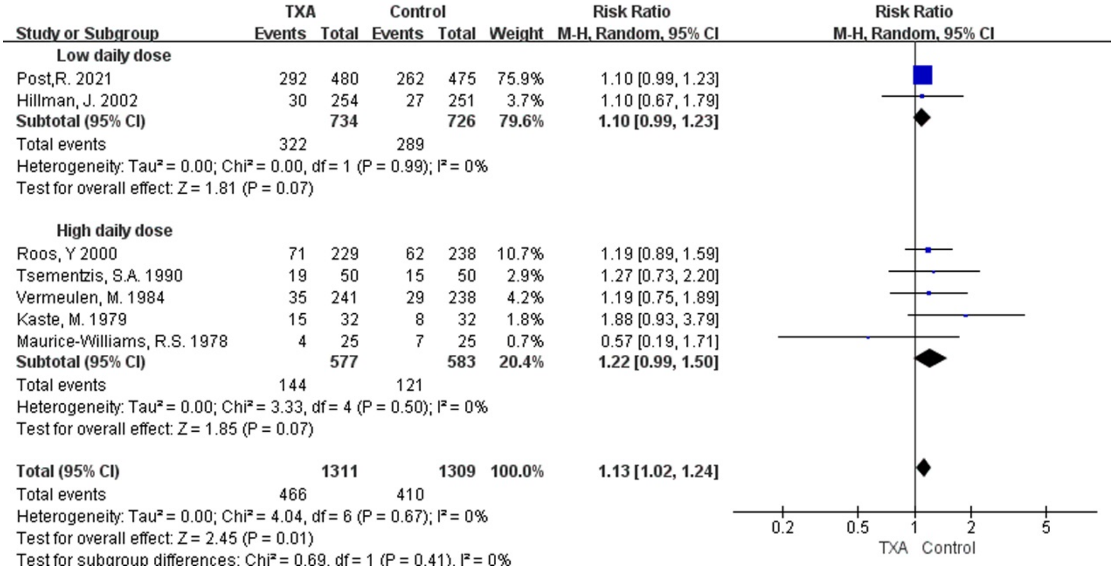

Supplementary figure 4. Forest plot of comparison in ***hydrocephalus****. CI = confidence interval, df = degrees of freedom, TXA = tranexamic acid, M-H = Mantel-Haenszel.*

Study or Subgroup	TXA Events	TXA Total	Control Events	Control Total	Weight	Risk Ratio M-H, Random, 95% CI
**Low daily dose**						
Post,R. 2021	108	480	106	475	29.0%	1.01 [0.80, 1.28]
Hillman, J. 2002	10	254	12	251	8.7%	0.82 [0.36, 1.87]
**Subtotal (95% CI)**		**734**		**726**	**37.7%**	**0.99 [0.79, 1.25]**
Total events	118		118			
Heterogeneity: Tau² = 0.00; Chi² = 0.22, df = 1 (P = 0.64); I² = 0%						
Test for overall effect: Z = 0.06 (P = 0.95)						
**High daily dose**						
Roos, Y 2000	68	229	74	233	27.0%	0.93 [0.71, 1.23]
Tsementzis, S.A. 1990	22	50	11	50	13.3%	2.00 [1.09, 3.68]
Vermeulen, M. 1984	59	241	36	238	22.0%	1.62 [1.11, 2.35]
**Subtotal (95% CI)**		**520**		**521**	**62.3%**	**1.38 [0.86, 2.21]**
Total events	149		121			
Heterogeneity: Tau² = 0.13; Chi² = 8.39, df = 2 (P = 0.02); I² = 76%						
Test for overall effect: Z = 1.34 (P = 0.18)						
**Total (95% CI)**		**1254**		**1247**	**100.0%**	**1.18 [0.89, 1.56]**
Total events	267		239			
Heterogeneity: Tau² = 0.05; Chi² = 10.17, df = 4 (P = 0.04); I² = 61%						
Test for overall effect: Z = 1.17 (P = 0.24)						
Test for subgroup differences: Chi² = 1.52, df = 1 (P = 0.22), I² = 34.1%						

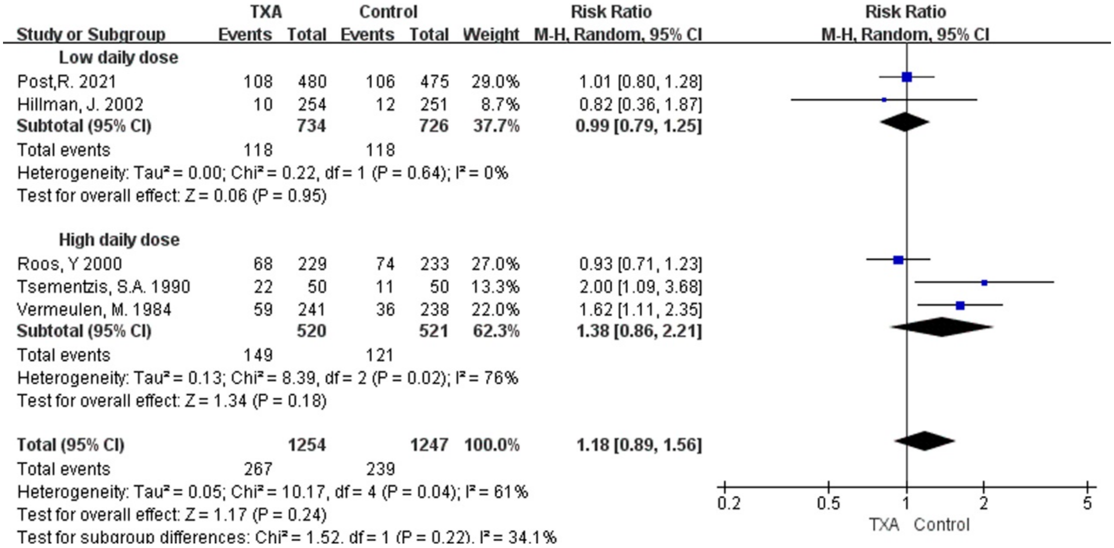

Supplementary figure 5. Forest plot of comparison in ***delayed cerebral ischemia****. CI = confidence interval, df = degrees of freedom, TXA = tranexamic acid, M-H = Mantel-Haenszel.*
